# Comprehensive ultrahigh resolution whole brain *in vivo* MRI dataset as a human phantom

**DOI:** 10.1038/s41597-021-00923-w

**Published:** 2021-05-25

**Authors:** Falk Lüsebrink, Hendrik Mattern, Renat Yakupov, Julio Acosta-Cabronero, Mohammad Ashtarayeh, Steffen Oeltze-Jafra, Oliver Speck

**Affiliations:** 1 Medicine and Digitalization, Department of Neurology, Medical Faculty, Otto-von-Guericke University, Magdeburg, Germany; 2 Biomedical Magnetic Resonance, Faculty of Natural Sciences, Otto-von-Guericke University, Magdeburg, Germany; 3 German Center of Neurodegenerative Diseases (DZNE), site Magdeburg, Germany; 4 Tenoke Ltd., Cambridge, UK; 5 Department of Systems Neuroscience, Center for Experimental Medicine, University Medical Center Hamburg-Eppendorf, Hamburg, Germany; 6 Center for Behavioral Brain Sciences, Magdeburg, Germany; 7 Leibniz Institute for Neurobiology, Magdeburg, Germany

**Keywords:** Imaging techniques, Diffusion tensor imaging, Brain imaging, Magnetic resonance imaging, Functional magnetic resonance imaging

## Abstract

Here, we present an extension to our previously published structural ultrahigh resolution T_1_-weighted magnetic resonance imaging (MRI) dataset with an isotropic resolution of 250 µm, consisting of multiple additional ultrahigh resolution contrasts. Included are up to 150 µm Time-of-Flight angiography, an updated 250 µm structural T_1_-weighted reconstruction, 330 µm quantitative susceptibility mapping, up to 450 µm structural T_2_-weighted imaging, 700 µm T_1_-weighted back-to-back scans, 800 µm diffusion tensor imaging, one hour continuous resting-state functional MRI with an isotropic spatial resolution of 1.8 mm as well as more than 120 other structural T_1_-weighted volumes together with multiple corresponding proton density weighted acquisitions collected over ten years. All data are from the same participant and were acquired on the same 7 T scanner. The repository contains the unprocessed data as well as (pre-)processing results. The data were acquired in multiple studies with individual goals. This is a unique and comprehensive collection comprising a “human phantom” dataset. Therefore, we compiled, processed, and structured the data, making them publicly available for further investigation.

## Abstract (summary)

**Table:** 

Measurement(s)	Diffusion Weighted Imaging • Magnetization-Prepared Rapid Gradient Echo MRI • Functional Magnetic Resonance Imaging • T2 (Observed)-Weighted Imaging • Gradient Echo MRI • MR Imaging Acquisition Protocol Element Time of Flight Contrast Indicator
Technology Type(s)	7 Tesla Magnetic Resonance Imaging
Sample Characteristic - Organism	Homo sapiens

Machine-accessible metadata file describing the reported data: 10.6084/m9.figshare.14095997

## Background & Summary

**Fig. 1:**
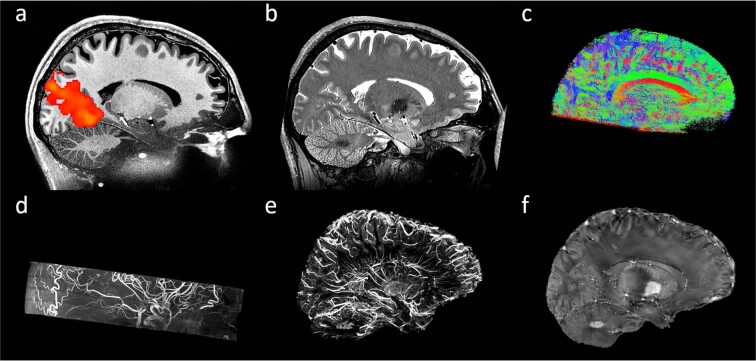
The human phantom. (**a**) 250 µm T_1_-weighted MPRAGE overlaid by 1.8 mm one hour rs-fMR. (**b**) 450 µm T_2_-weighted SPACE. (**c**) 800 µm DTI in twelve directions with a b-value of 750 s/mm². (**d**) 150 µm ToF angiography. (**e**) 330 µm venogram. (**f**) 330 µm QSM.

Previously, we published a human whole brain *in vivo* MRI dataset with an ultrahigh isotropic resolution of 250 µm^1^, freely available elsewhere^2,3^. It was very well received within the community and the number of downloads from the Dryad repository^2^ exceed 6,000 in total since publication in 2017. Now, we compiled data of the same participant acquired across more than ten years and present an extension to our previous dataset containing multiple additional contrasts with ultrahigh isotropic spatial resolution and (mostly) full brain coverage (Fig. 1). This includes up to 150 µm time of flight (ToF) angiography^4^, 250 µm MPRAGE^1^, 330 µm quantitative susceptibility mapping (QSM)^5^, up to 450 µm T_2_-weighted turbo spin echo (TSE), eight 700 µm T_1_-weighted back-to-back scans with low and high signal to noise ratio (SNR), 800 µm diffusion tensor imaging (DTI), one hour continuous 1.8 mm resting-state functional MRI (rs-fMRI), and more than 120 other MPRAGE volumes collected over 10 years with varying isotropic spatial resolution between 450 µm and 1 mm as well as many corresponding proton density weighted volumes with the same spatial resolution as the MPRAGE volumes. Thus, we have compiled the most comprehensive and high quality MRI data repository of a single human participant to date.

All data were acquired on the same 7 T scanner. Most of the data were acquired with prospective motion correction (MT384i, Metria Innovation Inc., WI, USA)^6^. The motion correction approach is considered the gold standard for high resolution imaging^7,8^. It allows unprecedented high resolution acquisitions as it prevents reduced effective resolution due to (unintentional) participant motion, thus overcomes the biological resolution limit^9^. The DTI and rs-fMRI data were corrected for geometric distortions by using a point-spread function approach^9,10^. With the exception of ToF and DTI, all acquisitions cover the entire brain. All non-MPRAGE data were rigidly registered to the previously published 250 µm MPRAGE using ANTs^11^ (available at: http://stnava.github.io/ANTs/). All longitudinally acquired MPRAGE data were quality checked with MRIQC^12^, biasfield corrected with SPM12^13^, and processed with the cross-sectional stream of FreeSurfer^14^, thus volumetry and cortical thickness measures alongside all segmentations (voxel- and surface-based) and many more outputs are readily available. The quantitative susceptibility maps (QSM)^5^ and QSM-based venogram were calculated using a self-written QSM pipeline^15^ and the DTI data were processed with TrackVis^16^. Based on the ToF data a maximum intensity projection was created to visualize the arteries. To ensure reusability, the data were structured according to BIDS^17^.

Apart for its use as a basis for an *in vivo* human brain atlas, the comprehensive high resolution human phantom may serve a number of further purposes: It allows for development and test of multimodal brain data fusion methodologies, methods that model temporal brain dynamics, complexity benchmark for data processing algorithms, structural and functional brain network modelling, brain connectivity analysis methods, classification using multimodal brain image data, or multimodal brain image visualization. The data may also be used for virtual teaching of brain anatomy.

Other publicly available datasets comprise data of a single participant from many years across different MR scanners and with multiple contrasts, e.g. SIMON^18^ or MyConnectome^19^. Compared to such longitudinal single participant studies, the MPRAGE data presented here is acquired on a single 7 T scanner without any hardware or software changes of the scanner, and mostly with prospective motion correction. The benefits of higher magnetic field strength results in either much higher SNR or higher resolution. Hence, this enables to study longitudinally volumetric changes in the (sub-)cortex with higher precision.

Due to its high resolution, structural, and vascular organization can be studied within (sub-) cortical regions to investigate potential interdependencies between arteries, veins, and gray matter. Further, the data can function as benchmark, e.g. Zhu *et al*.^20^ used the here presented high-resolution ToF and MPRAGE data as a reference to study the adaptation of the arterial supply in patients with glioblastoma.

The DTI and rs-fMRI data allow for joint connectivity analysis. Of the eight T_1_-weighted back-to-back scans, four were acquired with artificially low SNR by reducing the flip angle. Furthermore, the data is fully sampled. Hence, this dataset allows to study advanced reconstruction algorithms based on compressed sensing, denoising during reconstruction, or neural networks.

## Methods

The data were acquired from one healthy Caucasian male participant (born 1982) with no known history of cognitive impairment as well as neurodegenerative or psychiatric disease. The participant gave written informed consent prior to each individual study and, retrospectively, to share all data publicly. The local ethics committee has approved each study individually.

The data were collected from 2009 to 2020; across 66 scan sessions a total of 202 volumes have been acquired, of which 131 are MPRAGE acquisitions. All data were acquired on the same 7 T MR scanner (Siemens 7 T Classic, Siemens Healthineers, Erlangen, Germany) equipped with gradients of up to 70 mT/m per axis. From 2009 to 2011 data were acquired with a 1 Tx/24 Rx channel head coil (Nova Medical Inc, MA, USA) and from 2012 onwards with a 1 Tx/32 Rx channel head coil (Nova Medical Inc, MA, USA). The scanner’s software remained unchanged at VB17. Details of the sequences, protocols, and how each contrast was processed are given in Table 1 and their respective sections below.

**Table 1:** Brief sequence details.

Sequence	FoV, rounded [mm]	Iso. voxel size [mm]	TR/TE/TI [ms]	Undersampling/Accleration	Bandwidth [Hz/voxel]	ToA [hh:mm]/Measurements	Notes
T_1_ weighted MPRAGE	225 × 225 × 187	0.45	2820/2.82/1050	pFT (RO, Slc); GRAPPA 2	170	0:12/1	PMC
	224 × 224 × 157	0.7	2500/2.182/1050	None	430	1:44/8	PMC
T_2_ weighted TSE	200 × 200 × 158	0.45	5200/175/−	pFT (RO, Slc); GRAPPA 3; TurboFactor 173	531	0:22/1	PMC
	224 × 224 × 157	0.7	5500/133/−	pFT (RO, Slc); GRAPPA 3, TurboFactor 131	434	0:15/1	PMC
QSM 3D GRE	200 × 166 × 148	0.33	20/9.09/−	pFT (RO, Slc)	130	2:49/4	PMC
ToF 3D GRE	196 × 147 × 47	0.15	35/6.63/−	pFT (RO, PE, Slc)	102	2:14/1	PMC; MOTSA (4 Slabs, −25%); sSat
	196 × 147 × 78	0.25	50/6.63/−	pFT (RO, PE, Slc); GRAPPA 3	78	0:48:/1	PMC; MOTSA (4 Slabs, −25%); sSAT, MTC on
DTI 2D EPI	192 × 192 × 28	0.8	3560/(49;74)/−	pFT (PE); GRAPPA 2, TurboFactor 60	1096	3:40/4	FatSat; PSF DiCo; 4 Slabs; 12 directions (750 s/mm²)
rs-fMRI EPI	198 × 198 × 96	1.8	3080/20/−	pFT (PE); GRAPPA 4; TurboFactor 132	2104	1:02/1200	PMC, PSF DiCo; FatSat

For a full list of parameters, the exported protocols are included in the repository. pFT: Partial Fourier acquisition, RO: Readout direction, PE: Phase encoding direction, Slc: Slice direction, PMC: Prospective motion correction, PSF DiCo: Point spread function distortion correction, FatSat: Fat saturation, sSat: sparse saturation; MOTSA: multiple overlapping thin slab acquisition, MTC: magnetization transfer contrast.

### Prospective motion correction (PMC)

**Fig. 2:**
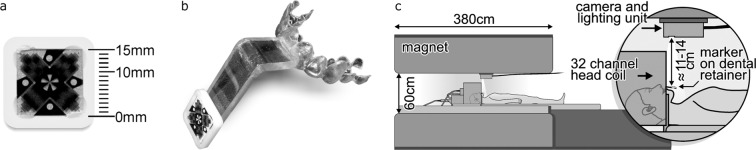
Setup for prospective motion correction using optical Moiré phase tracking. (**a**) Moiré phase tracking marker. (**b**) Individually manufactured mouthpiece with extension to protrude from the head coil. (**c**) Experimental setup for motion tracking during the MRI measurements. Reproduced from Stucht *et al*.^21^.

Most of the data compiled in this data descriptor have been acquired using prospective motion correction. To that end, a single, in-bore mounted camera tracks head motion with high precision and relatively high frequency in six degrees-of-freedom by capturing a single marker^6^ (Fig. 2a). The marker is attached to an individually crafted mouthpiece which rigidly connects the marker with the participant’s upper frontal teeth and, therefore, with the participant’s cranium^21^ (Fig. 2b). This approach allows tracking rigid motion of the brain. Even the rigid displacement of the head due to the breathing cycle and heartbeat can be tracked and corrected for, overcoming the biological resolution limit. However, non-rigid motion, e.g. due to pulsation of the ventricles or vessels cannot be corrected for using this approach. Further details can be found in the literature^4–6,21,22^.

### 150 µm & 250 µm Time-of-flight angiography

To prevent motion artefacts prospectively at 7 T a ToF sequence was implemented with PMC. To meet Specific Absorption Rate (SAR) constraints, venous saturation and magnetization transfer pulses where applied sparsely^23^. The implemented sparse saturation applied magnetization transfer pulses only during the acquisition of the central k-space (10% of all k-space) and applied venous saturation pulse every 7^th^ (250 µm) or 10^th^ (150 µm) excitation. Further, Variable-Rate Selective Excitation (VERSE)^24^ was used to reduce the amplitude of the venous saturation pulses. Sequence implementation details can be found elsewhere^4^. All ToF acquisitions used the Multiple Overlapping Thin Slab Acquisition (MOTSA) scheme^25^ and Tilted Optimized Nonsaturating Excitation (TONE) pulses^26^ to improve vessel depiction.

**Fig. 3:**
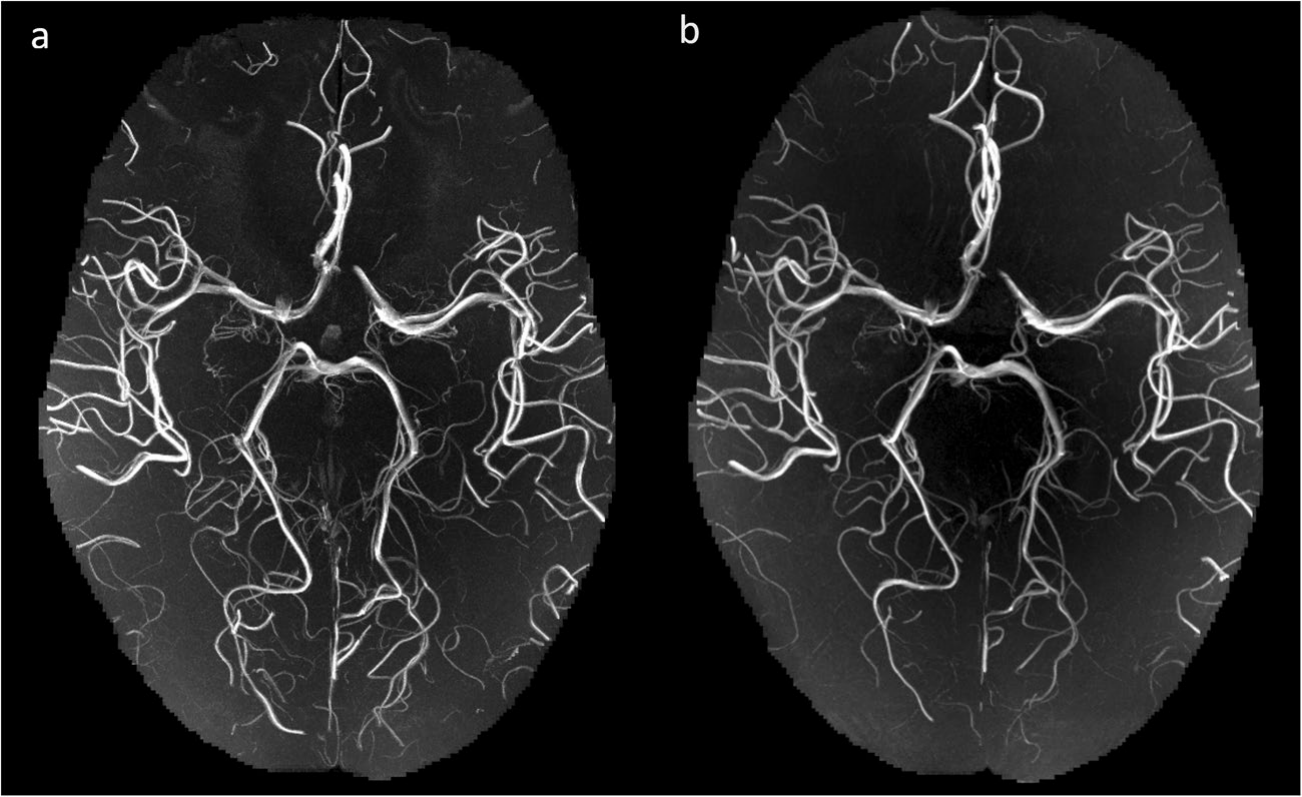
Maximum intensity projection of the time of flight angiography (axial view). MIP of slices 320 to 520 and identical windowing with a native isotropic resolution of (**a**) 150 µm and (**b**) 250 µm after registration to the 250 µm.

The higher resolution of the 150 µm data allows to depict arteries with much smaller diameter in the maximum intensity projection (Fig. 3). The 150 µm ToF data cover 46.8 mm in head-foot direction, missing most of the frontal and parietal lobes, as well as inferior parts of the temporal lobes and the entire cerebellum, while the 250 µm ToF data covers 78 mm of the brain in head-foot direction missing superior parts of the frontal and parietal lobes as well as inferior parts of the cerebellum. Further protocol details can be found in Table 1 and the full protocol can be found in the repository.

### 250 µm T1-weighted MPRAGE

**Fig. 4:**
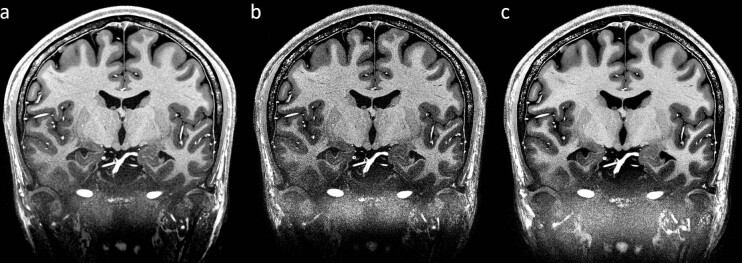
T_1_-weighted MPRAGE with an isotropic resolution of 250 µm based on eight repetitions. (**a**) Originally published version^1^ registered using a linear interpolator. (**b**) As before, but registered using a BSpline interpolator. The resulting image is much sharper, however, visually signal to noise ratio is considerably reduced. (**c**) Updated version based on the same raw data^3^, after application of a method to denoise during reconstruction^27^ and registration using a Bspline interpolator. Visually, in comparison to (**a**) the resulting image is much sharper while the overall image quality in terms of noise appears equal. The images are windowed identically.

**Fig. 5:**
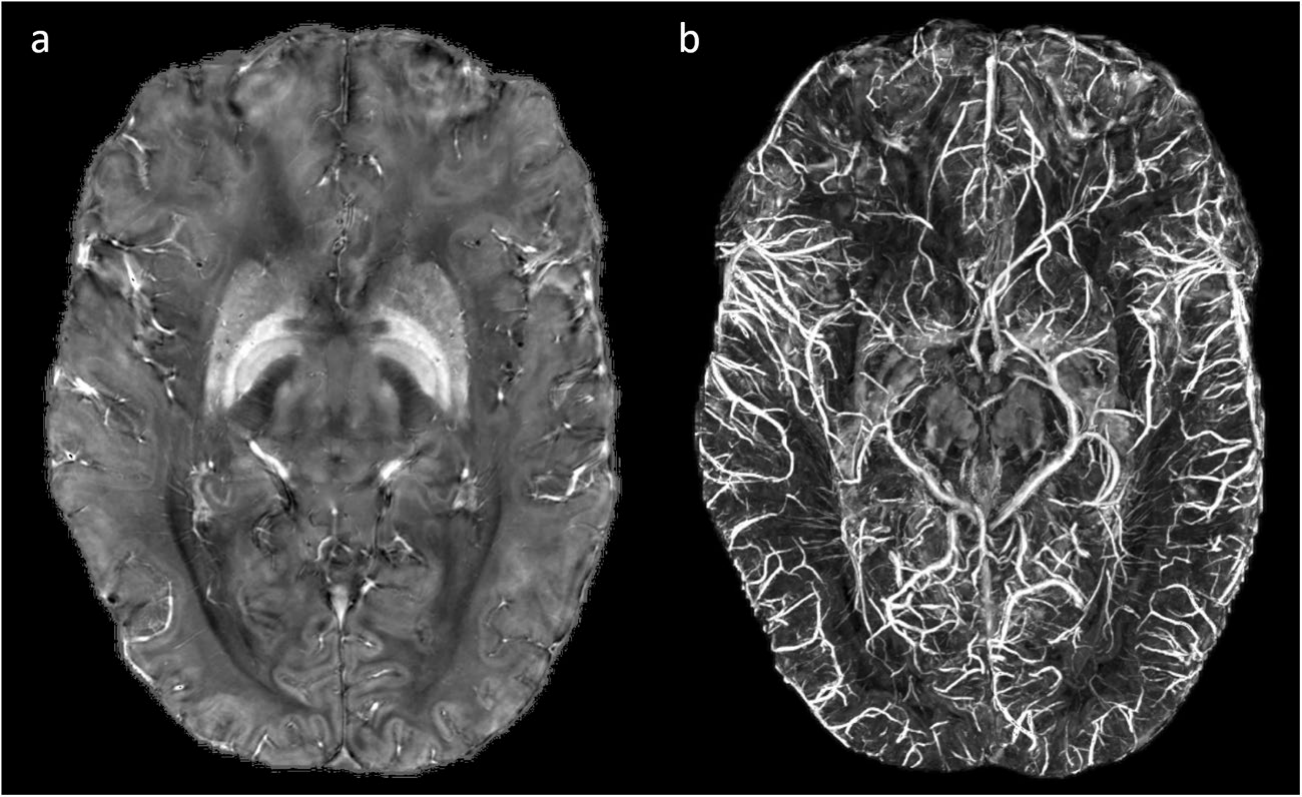
QSM with an isotropic resolution of 330 µm. (**a**) The level of detail allows for mapping of subcortical structures as well as susceptibility changes across the cortex allowing laminar QSM. (**b**) Maximum intensity projections of the QSM based venogram.

The data used in this section were published before^1–3^. The volume consists of eight repetitions, which were acquired in five different sessions across three months. The data were averaged after non-linear registration. Contrary to the previously published data, here, a forth order BSpline interpolation was used in the registration process instead of a linear interpolation method (Fig. 4a,b). This results in a much sharper depiction of details. However, SNR and, therefore, CNR, has decreased visually as less effective filtering occurred. In order to improve the signal to noise ratio, the scanner’s raw data^3^ were reconstructed offline using an in-house reconstruction pipeline with a newly established method to denoise during reconstruction on complex data per uncombined channel. The reconstruction pipeline was written in MATLAB and is publicly available on Github (https://www.github.com/fluese/reconstructionPipeline). Depending on the configuration of the denoising algorithms this enables to improve SNR, while preserving small details^27^. Here, the reconstruction pipeline has been set to compensate for the asymmetric echo as well as partial Fourier in slice direction by zero-filling. The Tukey filter to reduce Gibbs ringing was set to alpha equals 0.05. To denoise during reconstruction the BM4D^28^ filter was applied per uncombined channel with manually chosen very conservative settings^27^. The parameters of the reconstruction pipeline as well as the denoising method are compiled in a preset named ‘SciDataExt’ and can be selected from the options in the software. The resulting image (Fig. 5c) is visually as sharp as after BSpline interpolation (Fig. 4b), but the signal to noise ratio appears to be as good as after linear interpolation (Fig. 4a).

After the offline reconstruction, the data were biasfield corrected with SPM12 with medium regularization, a FWHM of 15, and a sampling distance of two. Otherwise, default parameters were chosen. Then the data were registered to an unbiased common template using ANTs to improve SNR by averaging using an adapted script especially for inter- and intrasession averaging of single participant data. The script is included in the repository called antsIntrasubjectAverage.sh. Subsequently, the data were registered to our previously published average created from the scanner’s reconstructed data^2^. To reduce interpolation artefacts, the transformations to the common template and previous average were applied in a single step. Both, the ANTs script to conduct the registration as well as the resulting transformations to perform the registration, are included in the repository. Therefore, the offline reconstruction can be compared to the previously published data based on the scanner’s reconstruction directly (Fig. 4). The biasfield correction with SPM12 can be applied within a function of the reconstruction pipeline and has been setup appropriately in the ‘SciDataExt’ preset. Furthermore, a standalone version of the script is available in the repository.

### 330 µm Quantitative Susceptibility Mapping

A spoiled Gradient-Recalled Echo (GRE) sequence with prospective motion correction was used to generate Quantitative Susceptibility Maps (QSM) with 330 µm isotropic resolution. Due to the high resolution, in total four averages have been acquired in two sessions on different days (two averages per session, acquisition time of each session 1:25 h). Magnitude and phase images, both coil combined (sum-of-squares) as well as uncombined, were exported from the scanner and are available in the repository.

The processing pipeline of the data is described in detail by Mattern *et al*.^5^. In brief, to compute QSM from the data (Fig. 5a), first a brain mask from the coil-combined magnitude images using BET^29^ (fractional threshold of 0.1; provided by FSL^30^) was generated, followed by zero-padding the uncombined phase data in all three dimensions by 100, 100, and 50 voxels, respectively. Next, HARPERELLA^31^ (10 mm convolving sphere radius, 200 iterations) was used to generate unwrapped, background field pre-filtered, and offset-normalized single channel phase data. Then, magnitude-weighted linear fitting was performed for pre-filtered phase combination across channels. Subsequently, finer removal of harmonic background fields with a spherical mean value filter using varying kernel sizes^32^ (kernel radius 25 mm at brain centroid; 1 mm at brain boundary) was conducted. Finally, susceptibility maps were computed using the ℓ1‐norm penalty‐based, morphology‐enabled, non‐linear dipole inversion (nMEDI) method (available from Cornell QSM toolbox: https://weill.cornell.edu/mri/pages/qsm) with dynamic model error reduction (MERIT)^33^ (Lagrange multiplier set to 1000). To generate venograms with this pipeline, the kernel radius of the spherical mean value filter was reduced to 3 mm. Hence, the data was spatially high-pass filtered to suppress large structures. Fine structures such as venous vasculature are unaffected by this filtering, resulting in QSM based venogram (Fig. 5b). A recently proposed QSM reconstruction pipeline (use_001[2]_def_msdi[2], https://gitlab.com/acostaj/QSMbox^15^) inspired by the concept of single-channel pre-filtering (extended for dipole inversion across multiple spatial scales) can be found in the public domain, which can be used to reproduce the susceptibility maps shown here.

Susceptibility maps were calculated separately for each session and were averaged after co-registering QSM from the second session to that from the first session. Transformation fields were estimated from co-registration of channel-combined magnitude images using ANTs with the antsRegistrationSyN.sh script performing rigid, affine, and deformable SyN registration in a multi‐resolution routine.

### 450 µm & 700 µm T2-weighted TSE

**Fig. 6:**
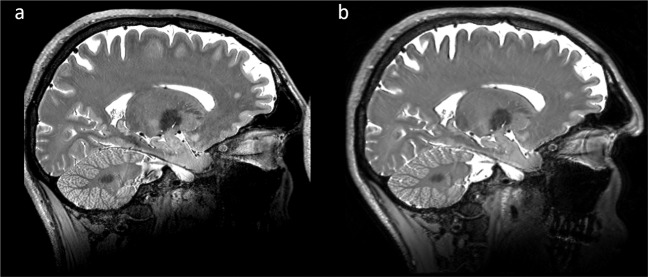
T_2_-weighted structural data. (**a**) Acquired using a SPACE sequence with an isotropic resolution of 450 µm and (**b**) acquired using a TSE sequence with an isotropic resolution of 700 µm.

To complement the structural data, we have included T_2_-weighted data to the repository with an isotropic resolution of 450 as well as 700 µm (Fig. 6). In the same session, we have collected T_1_-weighted datasets with the same resolution. Such additional contrast may be helpful for postprocessing procedures, segmentation algorithms, or manual localization of structures. The sequence parameters are briefly described in Table 1. All datasets were acquired using prospective motion correction to compensate for involuntary participant motion.

### 800 µm diffusion tensor imaging

The diffusion weighted echo planar images were obtained with a Stejskal-Tanner^34^ single shot spin echo EPI point spread function (PSF) sequence^9,10^. Due to the high spatial resolution of 800 µm the brain volume was divided into four slabs. Each slab was scanned in a separate session (cf. Table 1) as the time of acquisition per slab is close to one hour. The slabs do not cover inferior parts of the occipital and temporal lobes as well as only the cerebellum partly. In the first session, a MPRAGE with an isotropic resolution of 800 µm was acquired additionally as a structural reference. Each slab was measured thirteen times, once with b = 0 s/mm^2^ and for twelve diffusion directions with b = 750 s/mm^2^. The relatively low b-value of 750 s/mm^2^ is a tradeoff between acquisition time, image resolution, SNR, and diffusion weighting, among others. Each slab consists of 35 slices and was acquired in such way that slices of neighboring slabs overlap.

To combine the four slabs into a single volume the data were registered to the structural reference. However, before doing so, the different directions were motion corrected by rigid registration of the time series data to an unbiased template. To that end, we modified the multivariate template creation script of ANTs and included it in the repository as antsRegistrationSciData_averageDTI.sh. In a multistage rigid registration approach, an average volume from all directions was created and then each direction was registered to this unbiased average. This was done two times to receive a sharper average and, therefore, also more accurate motion correction.

After motion correction, the biasfield corrected structural volume from the first session was rigidly registered to the averaged slab from the motion correction using the antsRegistrationSciData.sh script with default parameters. The inverse of this registration transformed the slab into the space of the structural data. This registration yields more accurate results than registering the slab to the structural data and results inherently in padding of the volume, which allows for easier combination of the individual slabs later. To transform the DTI data into the space of the 250 µm structural data, the 800 µm structural data was rigidly registered to the 250 µm structural data using the antsRegistrationSciData.sh script with default parameters. Before that, the 250 µm data was downsampled to 800 µm to avoid upsampling the DTI. Subsequently, the forward transformation of the registration between the structural data and the inverse transformation of the structural to average DTI data was applied to the thirteen directions of the DTI data for each slab at once to avoid interpolation artefacts using antsApplyTransform with forth order BSpline interpolation.

**Fig. 7:**
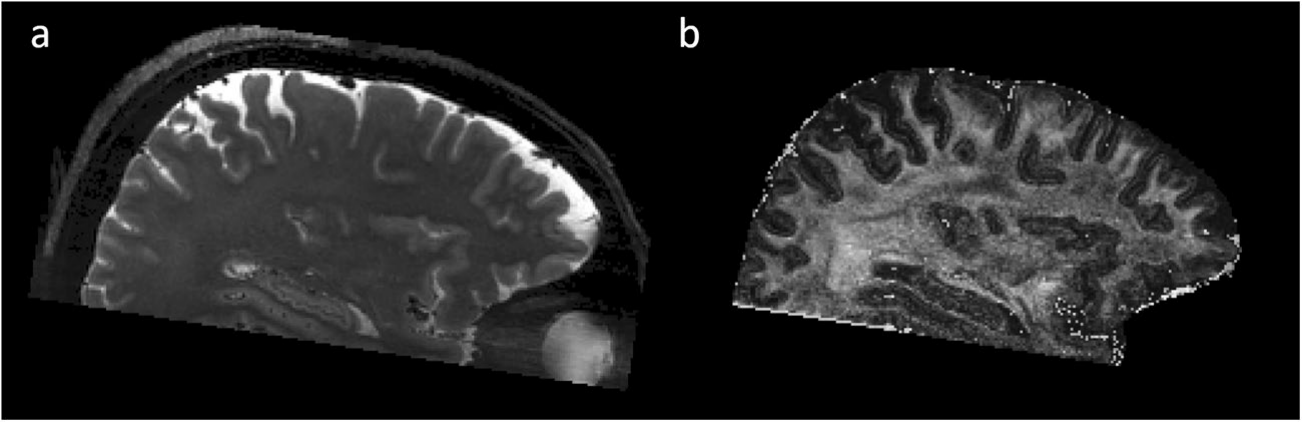
Diffusion tensor imaging. Four combined axial slabs of the (**a**) non-diffusion weighted image and (**b**) the fractional anisotropy map.

Finally, to combine the four slabs into a single volume, the weighted average of overlapping voxels of neighboring slabs was calculated relative to their distance. Prior to the weighting, the intensity of the slabs was normalized based on their white matter intensity to avoid intensity jumps between slabs. The MATLAB script including the entire procedure is included in the repository called weightedAverage_DTIslabs.m. After combining the slabs, we used TrackVis^16^ (http://www.trackvis.org) with following settings: Image model: DTI, maximum b value: 750, gradient table: siemens_12, angle threshold: 35, propagation algorithm: FACT, to generate the b_0_, diffusion weighted image, fractional anisotropy, ADC, eigenvalue and -vector maps, as well as the diffusion tensor data and tractography (Fig. 7).

### Back to back scans with high and low SNR

**Fig. 8:**
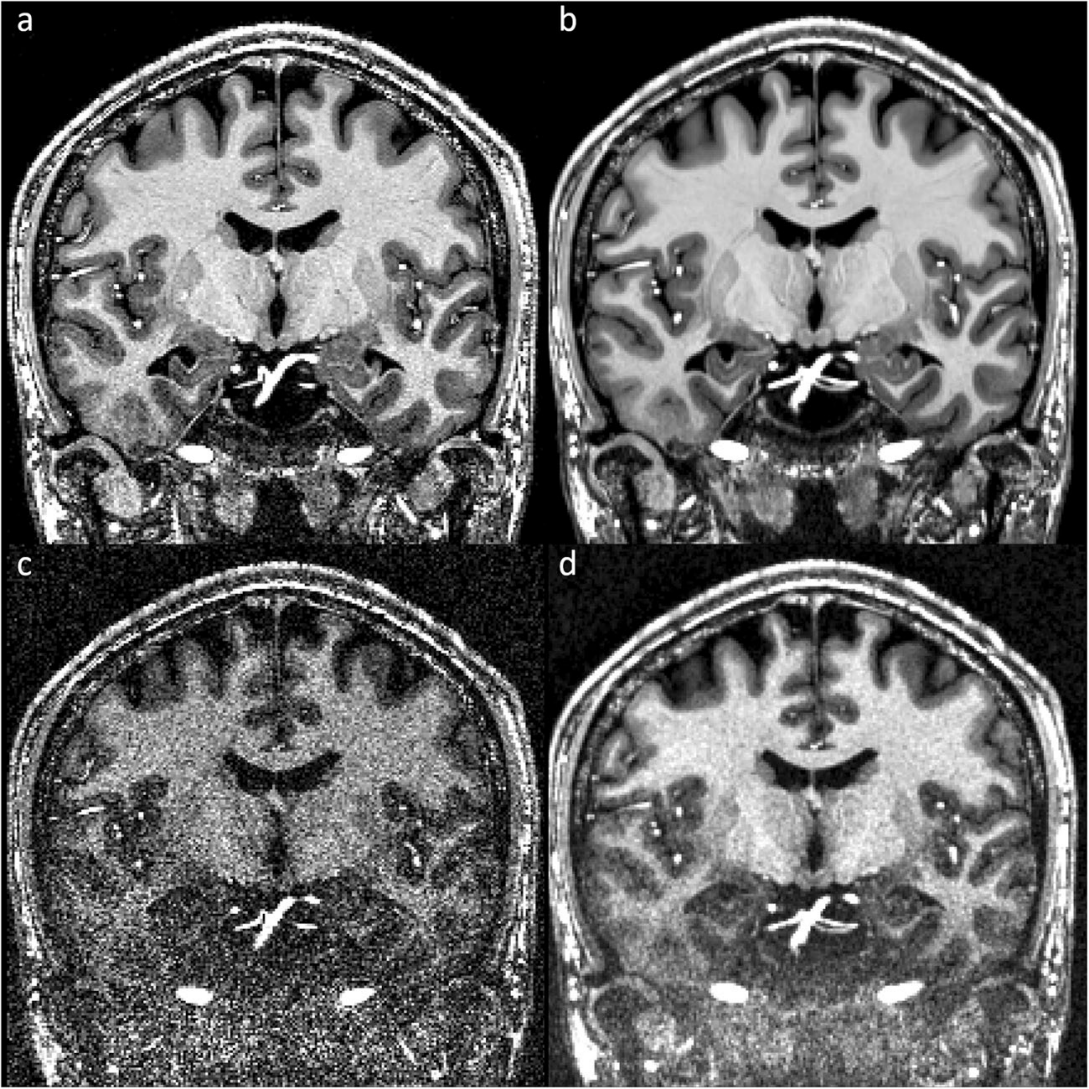
T_1_-weighted MPRAGE with an isotropic resolution of 700 µm of back-to-back scans. (**a**) Single high SNR acquisition. (**b**) Four averaged high SNR repetitions. (**c**) Single low SNR acquisition. (**d**) Four averaged low SNR repetitions.

In this subset of the data, eight volumes with an isotropic resolution of 700 µm were acquired back to back. All scans are fully sampled; no GRAPPA acceleration, no partial Fourier, and no asymmetric echo were applied. The first four repetitions were acquired with a flip angle of 5° to generate high SNR volumes, while the other four were acquired with a flip angle of 1° to generate artificially low SNR volumes (Fig. 8). A ninth volume with a flip angle of 0° was acquired additionally and, therefore, consists of noise only. All volumes were acquired using prospective motion correction. The most important sequence parameters are given in Table 1 briefly.

The data with high signal to noise ratio can be used to generate a reference dataset by initial registration of the individual volumes, followed by creating the median volume (Fig. 8b). Such dataset can for example be used as a reference for testing denoising algorithms on a single high SNR volume (Fig. 8a) or low SNR volumes (Fig. 8c,d). The registration has been conducted using an in-house registration script for ANTs, which is included in the repository and called antsIntrasubjectAverage.sh. This script allows for inter- and intrasession registration and averaging of data of the same participant.

In addition to the reconstructed image data, the scanners raw data are included in the repository. This allows for testing denoising algorithms, e.g. during reconstruction using the aforementioned reconstruction pipeline^27^. As the data are fully sampled, one can also undersample the data retrospectively, e.g. using a compressed sensing scheme and use the averaged high SNR data as reference for evaluation of the reconstruction.

### One hour continuous resting state functional MRI with 1.8 mm isotropic resolution

Resting-state fMRI data were acquired using a GE-EPI sequence with an isotropic resolution of 1.8 mm and one hour scan duration. In Table 1, the sequence parameters are briefly described. The data were prospectively motion-corrected^6^ and distortion-corrected online using the point-spread-function mapping approach^9^. Additionally, a MPRAGE with an isotropic resolution of 0.7 mm was acquired as structural reference in the same session. Alongside the imaging data, we have collected physiological data using a pulse oximeter (NONIN Puls Oxymeter 8600-FO) and breathing belt (Honeywell 40PC001B1A) which are included in the repository as well. The data is sampled at 200 Hz. The first row shows the respiration, the second the pulse, and the third the SpO2.

Both fMRI and T_1_-weighted MPRAGE data were re-oriented to standard space by means of FSL’s fslreorient2std routine. This was performed in order to help with subsequent co-registration steps. The T_1_-weighted MPRAGE data were segmented using SPM12 (bias regularization set to 0.0001, bias FWHM to 18 mm and clean up to thorough for improved segmentation of 7 T images^35^).

Temporal SNR (tSNR) was calculated as mean signal intensity divided by standard deviation over time for each voxel (both were calculated with FSL’s fslmaths routine). A brain mask was estimated from the T_1_-weighted MPRAGE scan as the sum of gray and white matter as well as CSF segmented images at 0.9 threshold. Holes in the mask were filled, and it was dilated twice, then eroded twice in order to smooth the mask boundary. These steps were also performed with FSL’s fslmaths routine. Mean tSNR estimated within this brain mask (warped into EPI space by means of ANTs – first, mean EPI image was rigidly co-registered to T_1_-weighted image with antsRegistrationSyNQuick, then the mask was registered back to EPI space with antsApplyTransforms) was 57.8, which is comparable to more common rs-fMRI acquisitions, despite considerably smaller voxel size (≈5 times smaller).

**Fig. 9:**
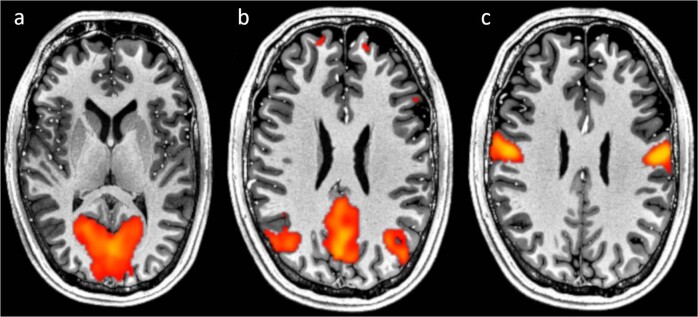
Examples of resting state networks. The networks are based on one-hour continuous rs-fMRI with an isotropic resolution of 1.8 mm after independent component analysis overlaid on 250 µm T_1_-weighed MPRAGE. (**a**) Visual component. (**b**) Default mode. (**c**) Auditory component.

FSL MELODIC independent component analysis was performed on the data with default parameters, other than no motion correction and limiting number of components to 33 (Fig. 9). All major resting-state components were observed with t-scores higher than 20.

### Structural longitudinal MPRAGE data

This dataset consists of 131 T_1_-weighted MPRAGE volumes acquired in 60 sessions from 2009 to 2020 with varying nominal isotropic resolution ranging from 450 µm to 1 mm. All volumes have been quality checked with MRIQC^12^, biasfield corrected with SPM12^13^ using parametrization suitable for a field strength of 7 T^35^, and processed with FreeSurfer^14^ using the HCP pipeline of version 6 with otherwise default parameters. This resamples the data to 1 mm isotropic; however, surface placement is done in native space. Hence, the surface segmentation is improved compared to the default processing stream especially in areas with high myelination, e.g. around the central sulcus^36^.

Alongside 34 of these volumes, data with PD-weighting were acquired in the same sessions to conduct biasfield correction by division^37^. The PD-weighted data were acquired using the MPRAGE sequence with the same parameters as the T_1_-weighted MPRAGE except for the inversion, which was removed and the repetition time, which was set to minimum (to mimic a FLASH sequence). In some cases, the PD-weighted volume was GRAPPA accelerated.

All data were acquired of the very same participant across more than 10 years on the same 7 T MRI in many different studies, e.g. for quantification of cortical thickness^38^, for setting up motion correction, for protocol testing, and so on. Therefore, the data are heterogeneous. The software version (VB17) of the scanner was identical throughout the entire time. From 2009 to 2011 a 1 Tx/ 24 Rx head coil was used and from 2011 onwards the same 1 Tx/ 32 Rx head coil was used in all studies. As described elsewhere^39–41^, the software version, head coil, field strength as well as other hardware components may have a significant impact on the data.

## Data Records

All data records listed in this section are hosted in the Open Science Repository of the library of the Otto-von-Guericke University, Magdeburg^42^ and are mirrored in the OpenNeuro repository^43^.

***Updated T***_***1***_***-weighted 250 µm dataset***

**Table:** 

**Original data**	/derivatives/sub-yv98/T1w_averages/sub-yv98_ses-3512 + 3555 + 3589 + 3637 + 3681_original_T1w.nii.gz
**File format**	NIfTI, gzip compressed
**Updated registration**	/derivatives/sub-yv98/T1w_averages/sub-yv98_ses-3512 + 3555 + 3589 + 3637 + 3681_BSpline_registration_T1w.gz
**File format**	NIfTI, gzip compressed
**Offline reconstructed data**	/derivatives/sub-yv98/T1w_averages/sub-yv98_ses-3512 + 3555 + 3589 + 3637 + 3681_offline_reconstruction_denoised-BM4D-manual_T1w.nii.gz
**File format**	NIfTI, gzip compressed
**Resulting data**	/derivatives/sub-yv98/registrations/sub-yv98_ses-3512 + 3555 + 3589 + 3637 + 3681_reconstructed_T1w_registered_to_sub-yv98_T1w_250um_original.nii.gz
**File format**	NIfTI, gzip compressed

This dataset contains the source data and derivatives of the T_1_ weighted data with an isotropic resolution of 250 µm. This includes the data after averaging the eight single repetitions. The data were registered non-linearly to a common unbiased template with ANTs.

***Ultrahigh resolution multi-contrast dataset***

**Table:** 

**ToF sourcedata**	/sourcadata/sub-yv98/ses-[3568, 3943]/anat/*.nii.gz
**File format**	NIfTI, gzip compressed
**ToF derivatives**	/derivatives/sub-yv98/ToF/*.tif
**File format**	TIF
**QSM sourcedata**	/sourcedata/sub-yv98/ses-[4088, 4137]/anat/*nii.gz
**File format**	NIfTI, gzip compressed
**QSM derivatives**	/derivatives/sub-yv98/QSM/sub-yv98_4088 + 4137_QSM*.nii.gz
**File format**	NIfTI, gzip compressed
**T2 sourcedata**	/sourcedata/sub-yv98/ses-[3777, 5233]/anat/*.nii.gz
**File format**	NIfTI, gzip compressed
**DTI sourcedata**	/derivatives/sub-yv98/ses-[4284, 4291, 4295, 4297]/dti/*.nii.gz
**File format**	NIfTI, gzip compressed
**DTI derivates**	/derivatives/sub-yv98/DTI/sub-yv98_ses-4284 + 4291 + 4295 + 4297_*.nii.gz
**File format**	NIfTI, gzip compressed
**rs-fMRI sourcedata**	/sourcedata/sub-yv98/ses-5124/fmri/*.nii.gz
**File format**	NIfTI, gzip compressed
**rs-fMRI physiodata**	/sourcedata/sub-yv98/ses-5124/physio/*.txt
**File format**	txt file
**rs-fMRI derivatives**	/derivatives/sub-yv98/rs-fMRI/sub-yv98_ses-5124*.nii.gz
**File format**	NIfTI, gzip compressed

This dataset contains the source data as well as derivatives of the ultrahigh resolution multi-contrast data, namely ToF (150 & 250 µm), QSM (330 µm), T_2_ (450 & 700 µm), DTI (800 µm), and rs-fMRI (1.8 mm).

***Registered ultrahigh resolution data***

**Table:** 

**Registered data**	/derivatives/sub-yv98/250um/registrations/*.nii.gz
**File format**	NIfTI, gzip compressed

This dataset contains the derivatives of the ultrahigh resolution multi-contrast data rigidly registered to the 250 µm data.

***Transformation of ultrahigh resolution registrations***

**Table:** 

**Transformation matrices**	/derivatives/sub-yv98/250um/transformations/*.mat
**File format**	mat file

This dataset contains the transformation files used for the registration of the derivatives of the ultrahigh resolution multi-contrast data to the 250 µm data.

T_1_-***weighted back-to-back scans***

**Table:** 

**High SNR data**	/sourcedata/sub-yv98/ses-4496/anat/sub-yv98_ses-4496_run-0[1, 2, 3, 4]_T1w.nii.gz
**File format**	NIfTI, gzip compressed
**Low SNR data**	/sourcedata/sub-yv98/ses-4496/anat/sub-yv98_ses-4496_run-0[5, 6, 7, 8]_T1w.nii.gz
**File format**	NIfTI, gzip compressed
**Noise data**	/sourcedata/sub-yv98/ses-4496/anat/sub-yv98_ses-4496_Noise.nii.gz
**File format**	NIfTI, gzip compressed

This dataset contains eight T_1_-weighted back-to-back scans using the MPRAGE sequence with an isotropic resolution of 700 µm. The first four scans have been acquired with a flip angle of 5°, while the next four acquisitions have been acquired with a flip angle of 1° to generate low SNR volumes. The four high SNR volumes have been registered and averaged to create a reference volume.

**Raw data of T**_**1**_**-*weighted back-to-back scans***

**Table:** 

**Raw data**	/rawdata/sub-yv98/ses-4496/meas*.dat
**File format**	dat files

This dataset contains the scanner’s raw data in Siemens file format of the T_1_-weighted back to back scans. This can be used to improve image reconstruction, e.g. by introducing methods like denoising during reconstruction.

***Longitudinal structural MPRAGE***

**Table:** 

**T1w sourcedata**	/sourcedata/sub-yv98/ses-*/*T1w.nii.gz
**File format**	NIfTI, gzip compressed

This dataset contains all 131 T_1_-weighted MPRAGE, 36 PD-weighted and 2 T_2_-weighted datasets acquired between 2009 and 2020 of the participant. The data was acquired at 7 T with the very same scanner and software version. From 2009 to 2011 a 24-channel head coil was used, and from 2012 onwards a 32-channel head coil.

***MRIQC of longitudinal MPRAGE***

**Table:** 

**MRIQC output**	/derivatives/sub-yv98/longitudinal_T1w/MRIQC/*.html
**File format**	HTML file

This dataset contains the MRIQC results of the T_1_-weighted longitudinal data.

***Biasfield corrections of longitudinal MPRAGE***

**Table:** 

**Biasfield corrected T1w data**	/derivatives/sub-yv98/ses-*/anat/sub-yv98_ses*_T1w_biasCorrected.nii.gz
**File format**	NIfTI, gzip compressed

This dataset contains the biasfield corrected T_1_-weighted longitudinal data using SPM12.

***FreeSurfer outputs of longitudinal MPRAGE***

**Table:** 

**FreeSurfer output**	/derivatives/sub-yv98/longitudinal_T1w/FreeSurfer/sub-yv98_ses-*_T1w_biasCorrected/*
**File format**	Various

This dataset contains the entire FreeSurfer v6 outputs of the biasfield corrected T_1_-weighted longitudinal data. This includes the voxel- and surface-based segmentations, volumetry results, cortical thickness measures, and many more outputs.

***Imaging protocols***

**Table:** 

**Imaging protocols**	/protocols/sub-yv98_ses-*.pdf
**File format**	PDF

This dataset contains the exported sequence protocols of the ultrahigh resolution multi-contrast data.

***Scripts***

**Table:** 

**Scripts**	/scripts/*
**File format**	Various

This dataset contains the modified scripts used to register the data with ANTs as well as the MATLAB scripts to combine the DTI slabs with weighted averaging and the biasfield correction script for SPM12.

## Technical Validation

In order to show the technical validity of the structural T_1_-weighted data, we ran MRIQC. MRIQC is an open source framework to assess the quality of MRI data without reference data. However, MRIQC cannot process any other modalities than T_1_-weighted, T_2_-weighted, and fMRI data as of yet. Therefore, the quality of the ToF angiography, QSM as well as DTI volumes cannot be assessed quantitatively with MRIQC. Nevertheless, we conducted basic processing of all datasets with standard processing tools indicating its high quality. The results are included in the repository as well. Furthermore, results of most of the included imaging data were published elsewhere^4,5^, showcasing their individual high quality. This is the first release of the entire data itself.

Besides the quality assessment of the longitudinally acquired T_1_-weighted MPRAGE data, the data were biasfield corrected with SPM12, and cross-sectionally quantified using FreeSurfer v6 running the HCP high resolution pipeline provided through an update available elsewhere (https://surfer.nmr.mgh.harvard.edu/pub/dist/freesurfer/6.0.0-patch/hcp/). Using the HCP pipeline the data is processed after resampling to 1 mm. However, the surfaces are fitted to the WM/GM as well as GM/CSF boundary in native resolution to make use of the natively higher resolution. This has shown to be advantageous in highly myelinated areas e.g. around the central sulcus where the measured thickness increases^36^. In all other areas, the measured cortical thickness decreases^36,44^. However, as a ground truth is missing, it can only be concluded, that the segmentation and, therefore, the measured thickness changes^38,44^. It is not possible to verify, whether this is a more accurate measure of the actual cortical depth^44^.

The methods of prospective motion correction as well as distortion correction have been published elsewhere^9,10^. The utilized technique for prospective motion correction is considered gold standard^7,8^.

## Usage Notes

Comprehensive and well documented data reduces the need for repeated studies and contributes to sustainable as well as reproducible science, especially when the data is acquired on a modality that is not widely available, such as motion corrected 7 T data. All data are entirely freely available without any constraints and are licensed under CC-0. As with our previously published data, the participant as well as we have waived all rights to the data where possible. Therefore, they can be used without limitations, even commercially. Nevertheless, the data repository as well as this publication should be cited.

We expect the data to be used in many multimodal processing schemes, e.g. data fusion for visualization as well as teaching, building of atlases, vascularization of subcortical structures such as the hippocampus, validation of connectivity models based on joint DTI and rs-fMRI data and many more. Due to its high resolution and high quality, we expect to see structures that were never identified before *in vivo*, in either of the contrasts. Further, structural and vascular differences in the laminar organization of the cortex could be investigated by assessing jointly the MPRAGE (cortical delineation), QSM (iron, myelin proxy; venous vasculature), ToF (arterial vasculature), and DTI (intra-cortical fiber tracts).

The provided as well as previously published scanner’s raw data have been offline reconstructed using our in-house self-written reconstruction pipeline. The pipeline as well as setting files are publicly available at: https://www.github.com/fluese/reconstructionPipeline. Nevertheless, we hope to encourage other groups to further improve image reconstruction. Based on our pipeline, we hope that denoising during reconstruction can be improved further by applying different kinds of noise filtering, e.g. neural networks trained on three dimensional MRI data instead of two dimensional natural scenery images or by applying well-tuned classical filters.

The more than 130 MPRAGE volumes collected over ten years can be used in different kinds of studies. First and foremost, they can be used to conduct a longitudinal study, e.g. with FreeSurfer or CAT12. This could for example indicate longitudinal changes across time. However, as the protocol is not identical for each measurement, this may reduce statistical performance. Nevertheless, this challenge opens up other possibilities, e.g. development of methods to retrospectively combine such different data. Furthermore, data is included in back to back studies within the very same session. This may allow for test-retest studies in software development. Beyond that, in the back to back studies data with artificially lower SNR are included by reducing the flip angle from 5° to 1°. These data can be used to validate denoising algorithms by using the averaged high SNR data as ground truth.

## Data Availability

The in-house reconstruction pipeline used to reconstruct the 250 µm data is publicly available on Github (https://github.com/fluese/reconstructionPipeline). For the QSM reconstructions an in-house pipeline was used and its successor is publically available (https://gitlab.com/acostaj/QSMbox). The ANTs scripts to create an unbiased interparticipant average volume used for the 250 µm and 700 µm datasets, the scripts to conduct the DTI pre-processing as well as the script used to register the data to the 250 µm dataset are included in the repository. Furthermore, the biasfield correction script of SPM12 is included in the repository as well.
